# Aortic valve implantation-induced conduction block as a framework towards a uniform electrocardiographic definition of left bundle branch block

**DOI:** 10.1007/s12471-021-01565-8

**Published:** 2021-04-30

**Authors:** S. Calle, M. Coeman, A. Demolder, T. Philipsen, P. Kayaert, M. De Buyzere, F. Timmermans, J. De Pooter

**Affiliations:** 1 Department of Cardiology, University Hospital Ghent, Ghent, Belgium; 2 Department of Cardiac Surgery, University Hospital Ghent, Ghent, Belgium

**Keywords:** Left bundle branch block, Transcatheter aortic valve replacement, Surgical aortic valve replacement, QRS notching

## Abstract

**Introduction:**

New-onset left bundle branch block (LBBB) following transcatheter or surgical aortic valve replacement (LBBB_AVI_) implies a proximal pathogenesis of LBBB. This study compares electrocardiographic characteristics and concordance with LBBB definitions between LBBB_AVI_ and non-procedure-induced LBBB controls (LBBB_control_).

**Methods:**

All LBBB_AVI_ patients at Ghent University Hospital between 2013 and 2019 were enrolled in the study. LBBB_AVI_ patients were matched for age, sex, ischaemic heart disease and ejection fraction to LBBB_control_ patients in a 1:2 ratio. For inclusion, a non-strict LBBB definition was used (QRS duration ≥ 120 ms, QS or rS in V1, absence of Q waves in V5-6). Electrocardiograms were digitally analysed and classified according to three LBBB definitions: European Society of Cardiology (ESC), Strauss and American Heart Association (AHA).

**Results:**

A total of 177 patients (59 LBBB_AVI_ and 118 LBBB_control_) were enrolled in the study. LBBB_AVI_ patients had more lateral QRS notching/slurring (100% vs 85%, *p* = 0.001), included a higher percentage with a QRS duration ≥ 130 ms (98% vs 86%, *p* = 0.007) and had a less leftward oriented QRS axis (−15° vs −30°, *p* = 0.013) compared to the LBBB_control_ group. ESC and Strauss criteria were fulfilled in 100% and 95% of LBBB_AVI_ patients, respectively, but only 18% met the AHA criteria. In LBBB_control_ patients, concordance with LBBB definitions was lower than in the LBBB_AVI_ group: ESC 85% (*p* = 0.001), Strauss 68% (*p* < 0.001) and AHA 7% (*p* = 0.035). No differences in electrocardiographic characterisation or concordance with LBBB definitions were observed between LBBB_AVI_ and LBBB_control_ patients with lateral QRS notching/slurring.

**Conclusion:**

Non-uniformity exists among current LBBB definitions concerning the detection of proximal LBBB. LBBB_AVI_ may provide a framework for more consensus on defining proximal LBBB.

**Supplementary Information:**

The online version of this article (10.1007/s12471-021-01565-8) contains supplementary material, which is available to authorized users.

## What’s new?

- Lateral QRS notching/slurring is an essential criterion for diagnosing proximal left bundle branch block (LBBB).
- The use of different LBBB definitions results in discordance when scoring LBBB.
- Aortic valve implantation (AVI)-induced LBBB showed the highest concordance with the 2013 European Society of Cardiology and Strauss definitions.
- AVI-induced LBBB provides a framework for uniform criteria for true proximal LBBB.

## Introduction

Left bundle branch block (LBBB) in humans was first recorded electrocardiographically in 1914 [1]. Multiple criteria for LBBB have been proposed since, based on experimental canine studies [1, 2], human case studies [3], electrophysiological data [4] and observations in cardiac resynchronisation therapy (CRT) responders [1, 5–7]. However, the electrocardiographic pattern of LBBB has not been fully clarified and various LBBB definitions are currently used [1, 8–10], resulting in significant discordance when scoring LBBB in clinical practice [7, 11].

Although conduction block may theoretically occur at any level in the His-Purkinje network, growing evidence suggests that only proximal left bundle branch (LBB) lesions cause ‘true’ LBBB [12] and that only ‘true’ LBBB is considered a strong predictor of CRT response in heart failure patients [6, 13]. Notching or slurring of the QRS complex during LBBB has been linked to a proximal origin of the LBB conduction block and might be considered a key feature of proximal LBBB [14, 15]. A limitation of current LBBB definitions is that they are not exclusively based on electrocardiographic observations in patients with proximal LBBB, which may contribute to the heterogeneity in LBBB definitions.

New-onset LBBB after transcatheter (TAVR) or surgical aortic valve replacement (SAVR) implies a proximal pathogenesis of LBBB and may provide a ‘framework’ towards uniform criteria for proximal LBBB. In this study, we compare the electrocardiographic characteristics and LBBB definitions in aortic valve implantation (AVI)-induced LBBB to a non-procedure-induced LBBB control group.

## Methods

### Study populations

Enrolled in the study were all patients with AVI-induced LBBB (LBBB_AVI_), including both patients with TAVR- and SAVR-induced LBBB, at Ghent University Hospital between January 2013 and June 2019. All patients with a primary TAVR and SAVR procedure and without pre-existing LBBB were screened. Exclusion criteria were pre-procedural ventricular pacing and peri-procedural permanent pacemaker implant. Presence of acute LBBB was scored within 24 h following TAVR/SAVR.

A control group of LBBB patients (LBBB_control_) consisted of randomly selected LBBB patients at Ghent University Hospital. LBBB_control_ patients were matched for age, sex, left ventricular ejection fraction (LVEF), history of coronary artery disease (CAD) and acute coronary syndrome to the LBBB_AVI_ group in a 2:1 ratio. In both the AVI and control groups, LBBB was defined according to broad conventional criteria (QRS duration (QRSD) ≥ 120 ms, QS or rS in lead V1 and absence of Q waves in leads V5 and V6) [16]. The study was approved by the ethics committee of Ghent University Hospital.

### Electrocardiographic analysis and LBBB definitions

Electrocardiograms (ECGs) were recorded at a sweep speed of 25 mm/s and a calibration of 10 mm/mV, and digitally stored in the MUSE ECG database (GE Healthcare, Chicago, IL, USA). All ECGs were independently reviewed by two investigators and classified according to three currently used LBBB definitions: European Society of Cardiology 2013 (ESC 2013) [9], Strauss et al. [1] and American Heart Association 2009 (AHA 2009) [10] (see Electronic Supplementary Material, Fig. S1). Continuous electrocardiographic characteristics (QRSD, QRS axis, R wave peak time (RWPT)) were digitally analysed by the Marquette 12SL algorithm (GE Healthcare) [17]. RWPT was defined according to the Minnesota Code [18].

### Validation of proposed criteria for proximal LBBB

Based on our observations in LBBB_AVI_, we adapted currently used LBBB criteria and propose a revised definition of LBBB. The revised definition was validated in consecutive LBBB patients (broad criteria) who underwent implantation of a CRT device at Ghent University Hospital according to current guidelines (LVEF ≤ 35%) [9] and who were categorised as CRT super-responders (LBBB_CRT_) based on improvement in LVEF to > 45% after at least 6 months of CRT therapy at a prospective echocardiographic examination between October 2018 and August 2020. All pre-implant ECGs were reviewed by an investigator blinded to the revised LBBB criteria.

### Statistical analysis

Categorical variables are expressed as absolute number (percentage). Continuous variables are expressed as mean (± standard deviation) in the case of Gaussian distribution or median (1st quartile; 3rd quartile) if data are non-Gaussian distributed. Normality was tested using the Shapiro-Wilk test. To compare means/medians of two variables, Student’s *t*-test and the Mann-Whitney U test were used. Comparison of categorical variables among groups was performed by Fisher’s exact test and chi-square test. Linear regression analysis was used to assess the effects of clinical, echo- and electrocardiographic parameters on QRSD. Statistical significance was set at a two-tailed probability level of < 0.05. All statistical analyses were performed using SPSS software (version 26.0, IBM, Armonk, NY, USA).

## Results

### Characteristics of patients with new-onset LBBB_AVI_

A total of 59 LBBB_AVI_ patients (34 TAVR and 25 SAVR patients, median age 82 years, 42% male) were enrolled in the study. The characteristics of the TAVR and SAVR patients are shown in the Electronic Supplementary Material (Table S1). All patients had severe aortic valve stenosis with an aortic valve area < 1.0 cm^2^ as the indication for TAVR/SAVR. Pre-procedural conduction disease (left anterior/posterior hemiblock or intraventricular conduction delay) was observed in 8 (15%) patients. All patients developed LBBB immediately during implantation or within 24 h post-procedure. Except for age, no significant differences were observed between TAVR and SAVR patients.

With the occurrence of LBBB_AVI_, QRSD increased from 94 (86;100) ms to 148 (140;160) ms (*p* < 0.001) (Tab. 1). An LBBB QRSD of ≥ 130 ms was observed in 98% of patients and, with regard to the Strauss definition, 95% of patients met the sex-specific QRSD cut-off (Tab. 2). Notably, 100% of females had a QRSD ≥ 130 ms and 88% of males had a QRSD ≥ 140 ms. QRSD in LBBB_AVI_ males was longer than in females (154 [145;162] ms vs 145 [138;153] ms, *p* = 0.006). No other electrocardiographic differences were observed between the sexes. In a multivariate linear regression model including age, height, weight, sex, CAD and end-diastolic diameter, only male sex was independently associated with increased QRSD (β = 11.49; *p* = 0.039). The baseline frontal QRS axis shifted from 9 (−15;45)° to −15 (−37;11)° post-AVI (*p* = 0.001). In 72% of LBBB_AVI_ patients with a normal QRS axis (90%), a leftward shift was observed.

**Table 1:** Clinical, echo- and electrocardiographic characteristics of aortic valve implantation (*AVI*)-induced left bundle branch block (*LBBB*_*AVI*_) and matched control LBBB (*LBBB*_*control*_) patients

			LBBB_AVI_ (*n* = 59)	LBBB_control_ (*n* = 118)	*p*-value
*Clinical characteristics*					
	Median age (years)		82 (75;85)	81 (75;84)	Matched
	Male		25 (42)	50 (42)	Matched
	BMI (kg/m^2^)		26 ± 4.5	26 ± 4.1	0.592
	BSA (m^2^)		1.80 ± 0.207	1.80 ± 0.207	0.920
	Coronary artery disease		23 (39)	46 (39)	Matched
	Acute coronary syndrome		6 (10)	12 (10)	Matched
*Echocardiographic measurements*					
	End-diastolic diameter (mm)		47 ± 6.0	48 ± 7.7	0.506
	Left ventricular mass/BSA (g/m^2^)		102 ± 28.9	103 ± 36.4	0.562
	Left ventricular systolic function				Matched
		Normal (≥ 55%)	41 (70)	82 (70)	
		Mildly reduced (45–54%)	9 (15)	18 (15)	
		Moderately reduced (30–44%)	7 (12)	14 (12)	
		Severely reduced (< 30%)	2 (3)	4 (3)	
*ECG measurements*					
	PR interval (ms)		191 (168;208)	174 (158;204)	0.072
	QRS duration (ms)		148 (140;160)	145 (136;154)	0.074
	Frontal QRS axis (°)		−15 (−37;11)	−30 (−45;−3)	** 0.013**
	R wave peak time (lead I) (µV)		58 (50;70)	62 (56;72)	0.065
	Notching/slurring lateral leads		59 (100)	100 (85)	** 0.001**
	Notching/slurring inferior leads		49 (83)	83 (70)	0.067
	Notching/slurring V1‑2		12 (20)	6 (5)	** 0.002**

Values are mean ± standard deviation, median (first quartile; third quartile) or number (%)

*BMI* body mass index, *BSA* body surface area

**Table 2:** Agreement with various left bundle branch block (*LBBB*) definitions among aortic valve implantation (*AVI*)-induced LBBB (*LBBB*_*AVI*_) and matched control LBBB patients (*LBBB*_*control*_)

		LBBB_AVI_ (*n* = 59)	LBBB_control_ (*n* = 118)	*p-*value
*LBBB definition features*				
	QRS duration ≥ 120 ms	59 (100)	118 (100)	NP
	QRS duration ≥ 130 ms	58 (98)	101 (86)	** 0.007**
	QRS duration ≥ 130 ms in females and ≥ 140 ms in males	56 (95)	95 (81)	** 0.012**
	QS or rS in V1	59 (100)	118 (100)	NP
	Absence of Q waves in V5‑6	59 (100)	118 (100)	NP
	Absence of Q waves in V5‑6 and I	57 (97)	111 (94)	0.720
	Absence of Q waves in V5‑6, I and aVL	50 (85)	92 (78)	0.286
	Presence of Q waves in aVL	9 (15)	26 (22)	0.286
	R wave peak time > 60 ms in V5‑6	16 (27)	20 (17)	0.113
	R wave peak time > 60 ms in V6 only	30 (51)	60 (51)	1.000
	Notching/slurring in V5‑6, I or aVL	59 (100)	100 (85)	** 0.001**
	Notching/slurring in V5‑6, I and aVL	32 (54)	37 (31)	** 0.003**
	Notching/slurring in ≥ 2 leads (I, aVL, V1‑2, V5-6)	59 (100)	93 (79)	**<** **0.001**
*LBBB definitions*				
	ESC 2013 definition	59 (100)	100 (85)	** 0.001**
	Strauss definition	56 (95)	80 (68)	**<** **0.001**
	AHA 2009 definition	10 (17)	8 (7)	** 0.035**
*AHA 2009 definition variations*				
	Absence of Q waves in V5-V6‑I and R wave peak time > 60 ms in V5‑6	10 (17)	8 (7)	** 0.035**
	Absence of Q waves in V5-V6-I-aVL and R wave peak time > 60 ms in V5‑6	10 (17)	8 (7)	** 0.035**
	Absence of Q waves in V5-V6‑I and no R wave peak time criterion	31 (53)	37 (31)	** 0.006**
	Absence of Q waves in V5-V6-I-aVL and no R wave peak time criterion	28 (48)	35 (30)	** 0.020**

Values are number (%)

*AHA* American Heart Association, *ESC* European Society of Cardiology, *NP* not possible

### Electrocardiographic analysis of LBBB_AVI_ and LBBB_control_

**Fig. 1:**
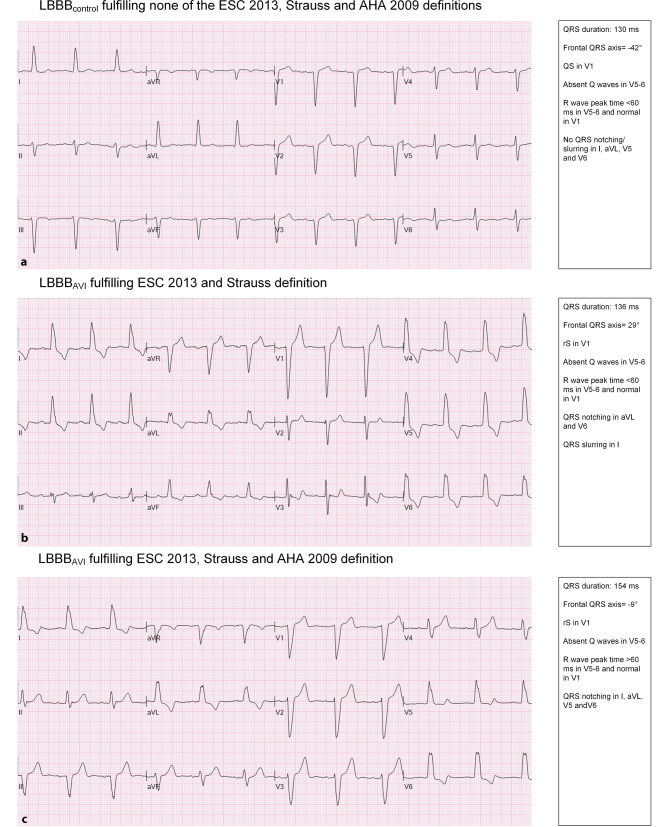
**a** Electrocardiogram of an 85-year-old control patient with non-procedure-induced left bundle branch block (*LBBB*_*control*_) fulfilling none of the European Society of Cardiology (*ESC*) 2013, Strauss and American Heart Association (*AHA*) 2009 definitions. **b** Electrocardiogram of a 72-year-old patient with left bundle branch block following aortic valve implantation (*LBBB*_*AVI*_) fulfilling the ESC 2013 and Strauss definitions. **c** Electrocardiogram of a 86-year-old LBBB_AVI_ patient fulfilling the ESC 2013, Strauss and AHA 2009 definitions

The 59 LBBB_AVI_ patients were matched to 118 LBBB_control_ patients. The characteristics of LBBB_AVI_ and LBBB_control_ patients are shown in Tab. 1. Representative ECGs are shown in Fig. 1. No clinical or echocardiographic differences were observed between the two groups.

All LBBB_AVI_ patients presented with QRS notching/slurring in the lateral leads (I, aVL, V5 or V6), whereas this was present in only 85% (100) of the LBBB_control_ group (*p* = 0.001). Inferior (lead II, III or aVF) and septal (lead V1 or V2) QRS notching/slurring was also more prevalent in the LBBB_AVI_ group (83% vs 70%, *p* = 0.067 and 20% vs 5%, *p* = 0.002, respectively).

Overall, QRSD was not significantly different between the two groups, neither was RWPT. However, patients with LBBB QRSD ≥ 130 ms (98% vs 86%, *p* = 0.007) and patients meeting the Strauss sex-specific QRSD cut-off (95% vs 81%, *p* = 0.012) were more frequently observed in the LBBB_AVI_ group.

### Classification according to current LBBB definitions

**Fig. 2:**
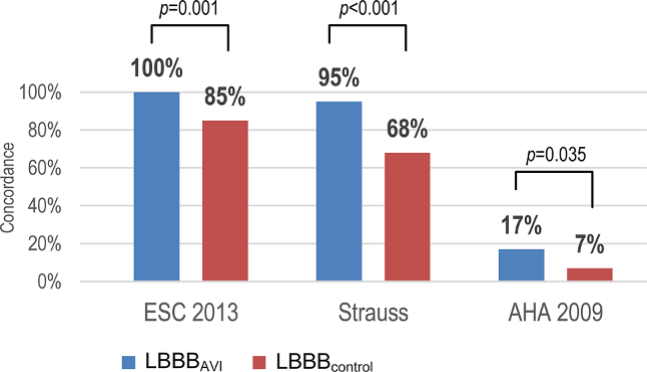
Classification according to currently used left bundle branch block (LBBB) definitions among aortic valve implantation-induced LBBB (*LBBB*_*AVI*_) patients and a non-procedure-induced LBBB control group (*LBBB*_*control*_)

Of all LBBB_AVI_ patients, 100% met the ESC 2013 and 95% the Strauss LBBB definition, whereas only 17% of patients met the AHA 2009 definition (Tab. 2, Fig. 2). Low concordance with the AHA definition is explained by the low prevalence of QRS notching/slurring combined in all four lateral leads (54%, Tab. 2). Interestingly, except for one patient, all LBBB_AVI_ patients had QRS notching in at least two lateral leads (I, aVL, V5 or V6). Furthermore, only 27% of patients had an RWPT > 60 ms in both leads V5 and V6, contributing to the low agreement with the AHA 2009 definition. When the analysis was restricted to the first three AHA 2009 criteria only, 48–53% of LBBB_AVI_ patients fulfilled the AHA 2009 definition. The presence of a Q wave in lead aVL minimally reduced adherence to the AHA 2009 definition (Tab. 2).

In the LBBB_control_ group, concordance with the different definitions was significantly lower than in the LBBB_AVI_ group: ESC 2013 85% (*p* = 0.001) and Strauss 68% (*p* < 0.001) (Tab. 2, Fig. 2). The lower agreement with the different LBBB definitions is explained by: (1) lower prevalence of lateral notching/slurring (85% vs 100%, *p* = 0.001) and (2) the higher number of patients with a shorter QRSD (QRSD ≥ 130 ms, 86% vs 98%, *p* = 0.007) in the LBBB_control_ group. Only 7% of patients fulfilled the AHA 2009 definition (*p* = 0.035, compared to LBBB_AVI_). As in the LBBB_AVI_ group, low concordance with the AHA 2009 criteria is caused by a low combined prevalence of QRS notching/slurring in all four lateral leads (31%) and most patients not meeting the RWPT criterion (83%).

### Subanalysis of LBBB_AVI_ versus LBBB_control_ with presence of lateral QRS notching

Comparison of LBBB_AVI_ patients and LBBB_control_ patients with lateral QRS notching/slurring is shown in the Electronic Supplementary Material (Table S2).

No differences in frontal QRS axis, nor in QRSD and number of patients with QRSD ≥ 130 ms were observed between the LBBB_AVI_ group and the LBBB_control_ group with lateral notching. Furthermore, concordance with the different LBBB definitions was comparable between LBBB_AVI_ patients and LBBB_control_ patients with lateral notching, but not when comparing LBBB_AVI_ versus LBBB_control_ without lateral notching.

### Extrapolation of LBBB_AVI_ features to LBBB_CRT_

Clinical, echo- and electrocardiographic characteristics of the 33 CRT responders (median age 61 (48;71) years, 52% male) are summarised in Tab. 3. During a median follow-up of 53 (20;77) months, as per definition, LVEF increased from 27 ± 5.9% to 54 ± 7.5% (*p* < 0.001). Except for an increased QRSD (160 [155;173] ms vs 148 [140;160] ms, *p* < 0.001) and a higher prevalence of QRS notching/slurring in all four lateral leads (76% vs 54%, *p* = 0.047) in the LBBB_CRT_ group, no differences in electrocardiographic features or agreement with the LBBB definitions were observed between LBBB_AVI_ and LBBB_CRT_.

**Table 3:** Baseline clinical, echo- and electrocardiographic characteristics of cardiac resynchronisation therapy (*CRT*) super-responders with left bundle branch block (*LBBB*) before CRT implantation

		LBBB_CRT_ (*n* = 33)	*p*-value compared to LBBB_AVI_
*Clinical characteristics*			
	Median age (years)	61 (48;71)	
	Male	17 (52)	
	Coronary artery disease	6 (18)	
	Acute coronary syndrome	1 (3)	
*Echocardiographic measurements*			
	End-diastolic diameter (mm)	61 ± 7.7	
	End-systolic diameter (mm)	52 ± 9.1	
	End-diastolic volume (ml)	185 ± 48.0	
	End-systolic volume (ml)	135 ± 37.0	
	Left ventricular ejection fraction (%)	27 ± 5.9	
*ECG measurements*			
	PR interval (ms)	180 (156;194)	0.103
	QRS duration (ms)	160 (155;173)	**<** **0.001**
	Frontal QRS axis (°)	−12 (−38;6)	0.937
*LBBB definition features*			
	QRS duration ≥ 120 ms	33 (100)	NP
	QRS duration ≥ 130 ms	32 (97)	0.149
	QRS duration ≥ 130 ms in females and ≥ 140 ms in males	31 (94)	0.369
	QS or rS in V1	33 (100)	NP
	Absence of Q waves in V5‑6	33 (100)	NP
	Notching/slurring in V5‑6, I or aVL	33 (100)	NP
	Notching/slurring in V5‑6, I and aVL	25 (76)	** 0.047**
	Notching/slurring in ≥ 2 leads (I, aVL, V1‑2, V5-6)	32 (97)	0.359
*LBBB definitions*			
	ESC 2013 definition	33 (100)	NP
	Strauss definition	32 (97)	1.000
	AHA 2009 definition	11 (33)	0.119
	AHA 2009 definition without R wave peak time criterion	23 (70)	0.127

Values are mean ± standard deviation, median (first quartile; third quartile) or number (%)

*AHA* American Heart Association, *ESC* European Society of Cardiology, *NP* not possible

## Discussion

### Main findings

This study assesses and reviews electrocardiographic features of LBBB in a population with proximal LBBB, i.e. patients with AVI-induced LBBB. As all LBBB_AVI_ patients had lateral QRS notching/slurring, QRS notching/slurring in the lateral leads is fundamental in the diagnosis of proximal LBBB. The LBBB_AVI_ group showed high concordance with ESC 2013 and Strauss definitions, but low agreement with the AHA 2009 definition. Our observations in LBBB_AVI_ were compared to matched LBBB patients from a general population, showing a higher number of patients with QRSD ≥ 130 ms, a higher prevalence of lateral QRS notching/slurring and a higher concordance with LBBB definitions in the LBBB_AVI_ group.

### Obstacles in defining LBBB

Current controversy in defining LBBB is primarily related to the difficulty in identifying patients with ‘true’ electrocardiographic LBBB. As studies over the past century have included patients with various types of conduction delay (proximal vs distal, focal vs diffuse), this obviously resulted in heterogeneous LBBB electrocardiographic patterns and criteria. Furthermore, most current LBBB definitions are derived from the same 1985 consensus criteria [19], but with different adaptations and interpretations (Electronic Supplementary Material, Fig. S1).

### True LBBB and proximal LBBB: two of a kind?

Although the importance of QRS notching/slurring was acknowledged even in early LBBB definitions [1, 20, 21], CRT was fundamental to the understanding of the relationship between electro-mechanical dyssynchrony in LBBB and a subset of LBBB electrocardiographic patterns with lateral QRS notching/slurring (‘true’ LBBB) [1, 13]. Patients without ‘true’ LBBB morphology were shown to demonstrate less electromechanical dyssynchrony [22], and absence of QRS notching/slurring resulted in less clinical and echocardiographic improvement than in true LBBB patients [6]. Experimental animal studies [14], His bundle pacing [23] and recent mapping studies [15] were able to link these ‘true’ LBBB electrocardiographic patterns with QRS notching to a proximal block in the LBB.

In our procedure-induced LBBB population, QRS notching/slurring was the most distinctive electrocardiographic characteristic of proximal LBBB. Our findings are in line with observational TAVR studies [24, 25] and a recent mapping study by Upadhyay et al., showing that QRS notching had the highest sensitivity and best negative predictive value to diagnose proximal LBBB [15]. Moreover, most proximal LBBBs were correctable by His bundle pacing in their study, indicating that in these patients no distal conduction disease was present. In contrast, LBBB patients without lateral notching demonstrated an intact proximal left conduction system and their LBBB was not correctable by His bundle pacing [15]. These findings suggest that an LBBB pattern without notching/slurring most likely reflects ‘distal block’ [1].

The question remains whether the presence of lateral QRS notching/slurring in a non-AVI-induced LBBB population also corresponds to proximal conduction disease of the left bundle. However, as uniformity was observed among LBBB_AVI_ patients and LBBB_control_ patients with lateral notching, these findings suggest pathophysiological similarities between the two groups and corroborate the evidence of a proximal block in all LBBB patients when notching/slurring is present.

### Proposed criteria for proximal LBBB

**Fig. 3:**
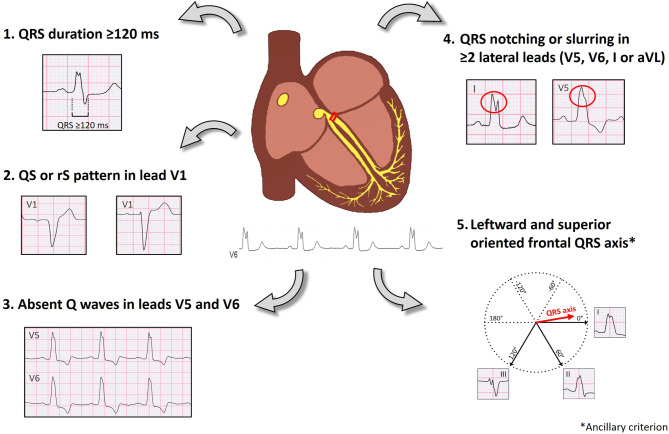
Proposed criteria for proximal left bundle branch block

AVI-induced LBBB implies an unequivocal proximal block and is therefore well suited for defining proximal LBBB. Based on our findings in LBBB_AVI_, we selected and adapted currently used criteria (Fig. 3).

#### QRS duration ≥120 ms

A minority of LBBB_AVI_ patients might present with a QRS duration <130 ms. Of interest is that female LBBB_AVI_ patients had a shorter QRSD than male LBBB_AVI_ patients. This is in line with the findings of previous work by our group, showing that female patients show proximal LBBB morphology at shorter QRSD [26].

#### QS or rS in lead V1 and absent Q waves in leads V5-6

At inclusion, all our patients had a QS or rS in lead V1 and absent Q waves in leads V5‑6. We observed a small Q wave in leads I and aVL in 15% of proximal LBBB patients and therefore recommend against an ‘absent Q wave’ criterion in leads I and aVL.

#### QRS notching or slurring in ≥ 2 lateral leads

Patients with proximal LBBB always presented QRS notching/slurring in at least two lateral leads. Only half of the LBBB_AVI_ patients had QRS notching/slurring in all four lateral leads, indicating that AHA 2009 requirements may lead to significant underdiagnosis of LBBB [7, 11, 27]. Whether the variable degree of QRS notching/slurring relates to suboptimal detection and/or differences in underlying electro-anatomical myocardial substrate remains unclear [28].

#### Leftward and superior oriented frontal QRS axis

Our observations in LBBB_AVI_ support those of previous studies [25, 29], which showed that the onset of LBBB causes a variable degree of QRS axis shift in a leftward and superior direction in most patients. A more leftward oriented QRS axis may support the diagnosis of proximal LBBB, but we recommend against absolute cut-off values because of the large range.

### Advantages of the revised LBBB definition

The ESC 2013 and Strauss definitions identify most LBBB_AVI_ patients. However, 5–14% of our proximal LBBB patients did not reach the computer-simulation-based Strauss QRSD thresholds of 130 and 140 ms for females and males, respectively. As such, QRS prolongation with a lower limit of 120 ms is preferable to define proximal LBBB. Although the ESC 2013 definition provides excellent sensitivity (100% of LBBB_AVI_ patients fulfilling the definition), the specificity might still be improved by adding the requirement of QRS notching/slurring in at least two lateral leads and the ancillary criterion of a leftward and superior oriented frontal QRS axis: 98% of LBBB_AVI_ patients had QRS notching/slurring in at least two lateral leads and 85% of patients had a QRS axis ≤ 30°. As AVI-induced LBBB patients and CRT super-responders both represent a ‘true’ LBBB electromechanical substrate within the large spectrum of left ventricular dysfunction, excellent compliance (97%) with the proposed criteria among CRT super-responders corroborates our revised LBBB definition.

### Limitations

A potential drawback in LBBB_AVI_ patients for studying the characteristics and definition of LBBB may relate to the age and co-morbidity in this particular population. Studying LBBB in a population with an unaffected myocardial substrate could overcome these issues. However, the almost identical observations in LBBB_AVI_, matched LBBB_control_ with lateral notching and LBBB_CRT_ patients argue against important myocardial substrate differences between these populations. In acute LBBB, as in LBBB_AVI_, electrical remodelling might affect electrocardiographic characteristics and alter LBBB features over time. However, this mainly involves changes in repolarisation features rather than changes in QRS features [30].

## Conclusion

In patients with proximal procedure-induced LBBB, the presence of QRS notching/slurring in the lateral leads seems a sine qua non for proximal LBBB. Non-uniformity exists among current recommendations for the diagnosis of proximal LBBB, with the ESC 2013 and Strauss definitions providing a higher sensitivity than the AHA 2009 definition. The LBBB_AVI_ population may therefore provide a framework for uniform criteria for assessing proximal LBBB.

## Supplementary Information

**Fig. S1 **A historical overview of electrocardiographic criteria for left bundle branch block [1, 8, 9, 10, 19, 20, 21]. Definitions and subsequent adaptations are connected with *arrows*. *QRSD* QRS duration, *RWPT* R wave peak time

**Table S1 **Baseline clinical, echo- and electrocardiographic characteristics of TAVR- and SAVR-induced LBBB patients

**Table S2 **Clinical, echo- and electrocardiographic characteristics of aortic valve implantation-induced LBBB and matched control LBBB patients (extended version)
