# Supplementary figures and images for: Aortic valve implantation-induced conduction block as a framework towards a uniform electrocardiographic definition of left bundle branch block
Source: Neth Heart J. 2021 Apr 30;29(12):643–53. doi: 10.1007/s12471-021-01565-8 (PMC8630173; doi:10.1007/s12471-021-01565-8)

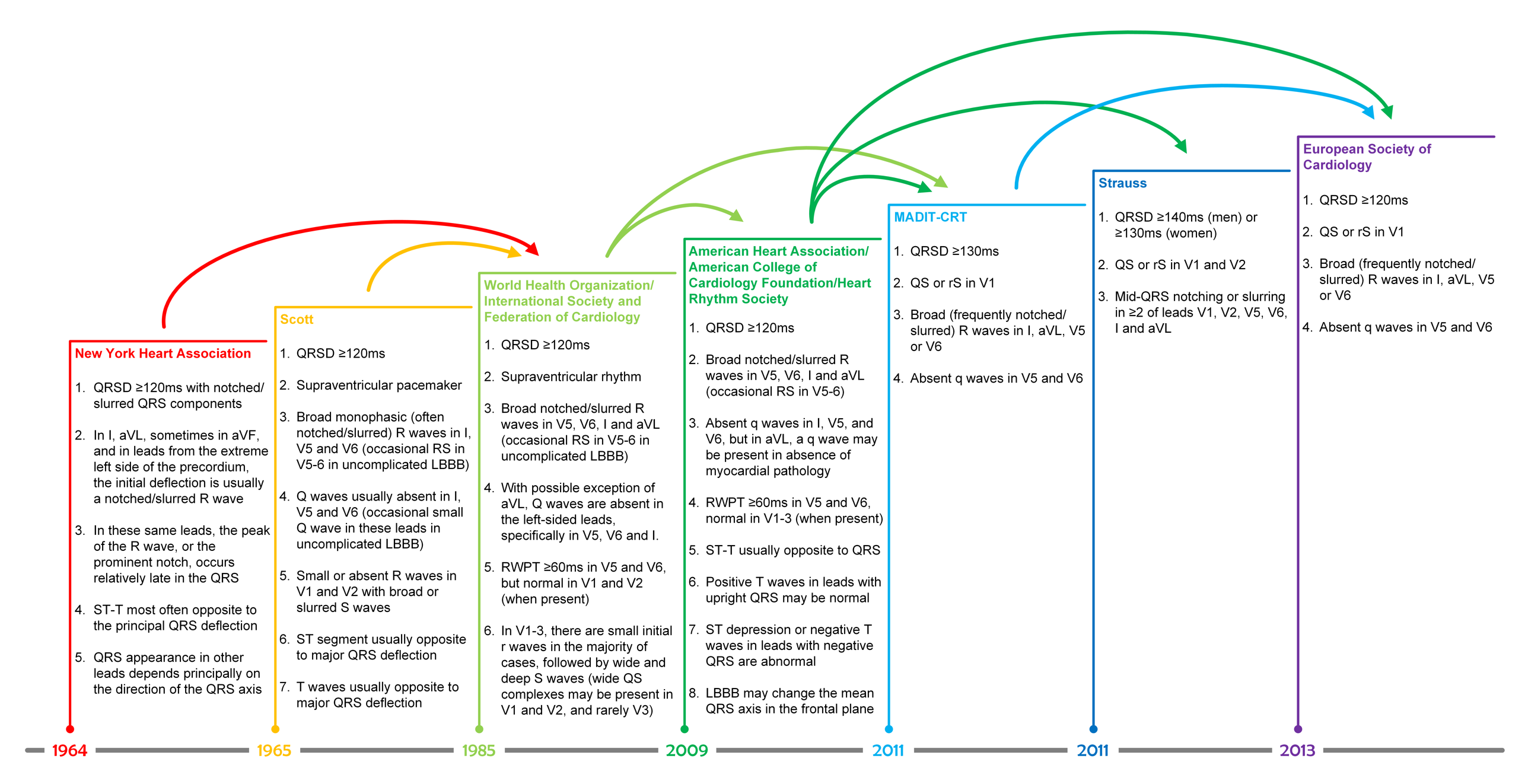

Supplement: Supplementary file 1 — Fig. S1 A historical overview of electrocardiographic criteria for left bundle branch block [1, 8, 9, 10, 19, 20, 21]. Definitions and subsequent adaptations are connected with arrows. QRSD QRS duration, RWPT R wave peak time [file 12471_2021_1565_MOESM1_ESM.png]
