# Supplementary material for: Aortic valve implantation-induced conduction block as a framework towards a uniform electrocardiographic definition of left bundle branch block
Source: Neth Heart J. 2021 Apr 30;29(12):643–53. doi: 10.1007/s12471-021-01565-8 (PMC8630173; doi:10.1007/s12471-021-01565-8)
Supplement: Supplementary file 2 — Table S1 Baseline clinical, echo- and electrocardiographic characteristics of TAVR- and SAVR-induced LBBB patients [file 12471_2021_1565_MOESM2_ESM.docx]

**Supplementary Table 1**

Baseline clinical, echo- and electrocardiographic characteristics of transcatheter aortic valve replacement (*TAVR*)- and surgical aortic valve replacement (*SAVR*)-induced left bundle branch block patients

|  |  | | **TAVR**  **(*n*=34)** | **SAVR**  **(*n*=25)** | ***p-*value** |
| --- | --- | --- | --- | --- | --- |
| **Clinical characteristics** | | |  |  |  |
|  | Median age (years) | | 84 (80;86) | 76 (65;80) | **<0.001** |
|  | Male | | 14 (41) | 11 (44) | 0.828 |
|  | BMI (kg/m²) | | 26±4.7 | 27±4.2 | 0.457 |
|  | BSA (m²) | | 1.77±0.200 | 1.83±0.215 | 0.353 |
| **Underlying heart disease** | | |  |  |  |
|  | Coronary artery disease | | 11 (32) | 12 (48) | 0.223 |
|  | Acute coronary syndrome | | 5 (15) | 1 (4) | 0.184 |
| **Echocardiographic measurements** | | |  |  |  |
|  | End-diastolic diameter (mm) | | 47±5.5 | 46±6.8 | 0.759 |
|  | Left ventricular mass/BSA (g/m²) | | 100±30.3 | 106±27.2 | 0.462 |
|  | Left ventricular systolic function | |  |  | 0.051 |
|  |  | Normal (≥55%) | 22 (65) | 19 (76) |  |
|  |  | Mildly reduced (45–54%) | 8 (24) | 1 (4) |  |
|  |  | Moderately reduced (30–44%) | 2 (6) | 5 (20) |  |
|  |  | Severely reduced (<30%) | 2 (6) | 0 (0) |  |
| **ECG measurements** | | |  |  |  |
|  | PR interval (ms) | | 189 (173;225) | 192 (161;205) | 0.516 |
|  | QRS duration (ms) | | 145 (138;162) | 150 (144;155) | 0.667 |
|  | Frontal QRS axis (°) | | -16 (-42;9) | -14 (-30;24) | 0.438 |
|  | R wave peak time (lead I) (µV) | | 60 (52;70) | 54 (50;74) | 0.401 |
|  | QT (ms) | | 465 (440;485) | 444 (420;472) | 0.068 |
|  | Notching/slurring lateral leads | | 34 (100) | 25 (100) | NP |
|  | Notching/slurring inferior leads | | 28 (82) | 21 (84) | 1.000 |
|  | Notching/slurring V1-2 | | 5 (15) | 7 (28) | 0.327 |
|  | | | | | |
| Values are mean ±standard deviation, median (first quartile; third quartile) or number (%) | | | | | |
|  | | | | | |
| *BMI* body mass index, *BSA* body surface area, *LBBB* left bundle branch block, *SAVR* surgical aortic valve replacement, *TAVR* transcatheter aortic valve replacement | | | | | |
