# Supplementary material for: Aortic valve implantation-induced conduction block as a framework towards a uniform electrocardiographic definition of left bundle branch block
Source: Neth Heart J. 2021 Apr 30;29(12):643–53. doi: 10.1007/s12471-021-01565-8 (PMC8630173; doi:10.1007/s12471-021-01565-8)
Supplement: Supplementary file 3 — Table S2 Clinical, echo- and electrocardiographic characteristics of aortic valve implantation-induced LBBB and matched control LBBB patients (extended version) [file 12471_2021_1565_MOESM3_ESM.docx]

**Supplementary Table 2**

Clinical, echo- and electrocardiographic characteristics of aortic valve implantation (*AVI*)-induced left bundle branch block (*LBBB_AVI_*) and matched control LBBB (*LBBB_control_*) patients (extended version)

|  |  | | **LBBB_AVI_**  **(*n*=59)** | **LBBB_control_**  **(*n*=118)** | ***p*-value*** | **LBBB_control_ with notching**  **(*n*=100)** | ***p*-value*** | **LBBB_control_ without notching**  **(*n*=18)** | ***p-*value*** |
| --- | --- | --- | --- | --- | --- | --- | --- | --- | --- |
| **Clinical characteristics** | | |  |  |  |  |  |  |  |
|  | Median age (years) | | 82 (75;85) | 81 (75;84) | matched | 81 (75;84) | 0.888 | 82 (73;84) | 0.768 |
|  | Male | | 25 (42) | 50 (42) | matched | 40 (40) | 0.769 | 10 (56) | 0.325 |
|  | BMI (kg/m²) | | 26±4.5 | 26±4.1 | 0.592 | 26±4.0 | 0.310 | 28±4.2 | 0.215 |
|  | BSA (m²) | | 1.80±0.207 | 1.80±0.207 | 0.920 | 1.78±0.187 | 0.474 | 1.94±0.254 | 0.061 |
|  | Coronary artery disease | | 23 (39) | 46 (39) | matched | 37 (37) | 0.803 | 9 (50) | 0.406 |
|  | Acute coronary syndrome | | 6 (10) | 12 (10) | matched | 10 (10) | 0.973 | 2 (11) | 1.000 |
| **Echocardiographic measurements** | | |  |  |  |  |  |  |  |
|  | End-diastolic diameter (mm) | | 47±6.0 | 48±7.7 | 0.506 | 48±7.8 | 0.890 | 50±7.1 | 0.075 |
|  | Left ventricular mass/BSA (g/m²) | | 102±28.9 | 103±36.4 | 0.562 | 102±37.2 | 0.386 | 107±34.0 | 0.782 |
|  | Left ventricular systolic function | |  |  | matched |  | 0.954 |  | 0.210 |
|  |  | Normal (≥55%) | 41 (70) | 82 (70) |  | 68 (68) |  | 14 (78) |  |
|  |  | Mildly reduced (45–54%) | 9 (15) | 18 (15) |  | 18 (18) |  | 0 (0) |  |
|  |  | Moderately reduced (30–44%) | 7 (12) | 14 (12) |  | 10 (10) |  | 4 (22) |  |
|  |  | Severely reduced (<30%) | 2 (3) | 4 (3) |  | 4 (4) |  | 0 (0) |  |
| **ECG measurements** | | |  |  |  |  |  |  |  |
|  | PR interval (ms) | | 191 (168;208) | 174 (158;204) | 0.072 | 173 (158;206) | 0.067 | 178 (168;198) | 0.428 |
|  | QRS duration (ms) | | 148 (140;160) | 145 (136;154) | 0.074 | 146 (138;154) | 0.305 | 133 (126;149) | **0.001** |
|  | Frontal QRS axis (°) | | -15 (-37;11) | -30 (-45;-3) | **0.013** | -28 (-44;4) | 0.082 | -37 (-51;-31) | **<0.001** |
|  | R wave peak time (lead I) (µV) | | 58 (50;70) | 62 (56;72) | 0.065 | 62 (56;74) | **0.044** | 58 (54;68) | 0.736 |
|  | Notching/slurring lateral leads | | 59 (100) | 100 (85) | **0.001** | 100 (100) | NP | 0 (0) |  |
|  | Notching/slurring inferior leads | | 49 (83) | 83 (70) | 0.067 | 83 (83) | 0.993 | 0 (0) |  |
|  | Notching/slurring V1-2 | | 12 (20) | 6 (5) | **0.002** | 6 (6) | **0.009** | 0 (0) |  |
| **LBBB definition features** | | |  |  |  |  |  |  |  |
|  | QRS duration ≥120 ms | | 59 (100) | 118 (100) | NP | 100 (100) | NP | 18 (100) | NP |
|  | QRS duration ≥130 ms | | 58 (98) | 101 (86) | **0.007** | 90 (90) | 0.055 | 11 (61) | **<0.001** |
|  | QRS duration ≥130 ms in females and ≥140 ms in males | | 56 (95) | 95 (81) | **0.012** | 86 (86) | 0.111 | 9 (50) | **<0.001** |
|  | QS or rS in V1 | | 59 (100) | 118 (100) | NP | 100 (100) | NP | 18 (100) | NP |
|  | Absence of q waves in V5-6 | | 59 (100) | 118 (100) | NP | 100 (100) | NP | 18 (100) | NP |
|  | Absence of q waves in V5-6 and I | | 57 (97) | 111 (94) | 0.720 | 97 (97) | 1.000 | 14 (78) | **0.024** |
|  | Absence of q waves in V5-6, I and aVL | | 50 (85) | 92 (78) | 0.286 | 85 (85) | 0.965 | 7 (39) | **<0.001** |
|  | Presence of q waves in aVL | | 9 (15) | 26 (22) | 0.286 | 15 (15) | 0.965 | 11 (61) | **<0.001** |
|  | R wave peak time >60 ms in V5-6 | | 16 (27) | 20 (17) | 0.113 | 19 (19) | 0.233 | 1 (6) | 0.060 |
|  | R wave peak time >60 ms in V6 only | | 30 (51) | 60 (51) | 1.000 | 59 (59) | 0.317 | 1 (6) | **0.001** |
|  | Notching/slurring in V5-6, I or aVL | | 59 (100) | 100 (85) | **0.001** | 100 (100) | NP | 0 (0) |  |
|  | Notching/slurring in V5-6, I and aVL | | 32 (54) | 37 (31) | **0.003** | 37 (37) | **0.034** | 0 (0) |  |
|  | Notching/slurring in ≥2 leads (I, aVL, V1-2, V5-6) | | 59 (100) | 93 (79) | **<0.001** | 93 (93) | **0.047** | 0 (0) |  |
| **LBBB definitions** | | |  |  |  |  |  |  |  |
|  | ESC 2013 definition | | 59 (100) | 100 (85) | **0.001** | 100 (100) | NP | 0 (0) | **<0.001** |
|  | Strauss definition | | 56 (95) | 80 (68) | **<0.001** | 80 (80) | 0.10 | 0 (0) | **<0.001** |
|  | AHA 2009 definition | | 10 (17) | 8 (7) | **0.035** | 8 (8) | 0.085 | 0 (0) | 0.061 |
| **AHA 2009 definition variations** | | |  |  |  |  |  |  |  |
|  | Absence of q waves in V5-V6-I and R wave peak time >60 ms in V5-V6 | | 10 (17) | 8 (7) | **0.035** | 8 (8) | 0.085 | 0 (0) | 0.061 |
|  | Absence of q waves in V5-V6-I-aVL and R wave peak time >60 ms in V5-V6 | | 10 (17) | 8 (7) | **0.035** | 8 (8) | 0.085 | 0 (0) | 0.061 |
|  | Absence of q waves in V5-V6-I and no R wave peak time criterion | | 31 (53) | 37 (31) | **0.006** | 37 (37) | 0.056 | 0 (0) | **<0.001** |
|  | Absence of q waves in V5-V6-I-aVL and no R wave peak time criterion | | 28 (48) | 35 (30) | **0.020** | 35 (35) | 0.121 | 0 (0) | **<0.001** |
|  | | | | | | | | | |
| *p*-values comparing LBBB_control_, LBBB_control_ with notching and LBBB_control_ without notching to LBBB_AVI_ are marked with an asterisk (*) | | | | | | | | | |
|  | | | | | | | | | |
| Values are mean ± standard deviation, median (first quartile; third quartile) or number (%) | | | | | | | | | |
|  | | | | | | | | | |
| *AVI* aortic valve implantation, *AHA* American Heart Association, *BMI* body mass index, *BSA* body surface area, *ESC* European Society of Cardiology, *LBBB* left bundle branch block | | | | | | | | | |
